# Agronomic Performance, Mineral Composition, and Biochemical Characteristics of Basil (*Ocimum basilicum* L.) Grown in Trout (*Oncorhynchus mykiss*) Aquaponic Systems

**DOI:** 10.3390/biology15060511

**Published:** 2026-03-22

**Authors:** Mohammed Elakrouch, Marouane Mohaddab, Sarah Elmoussaoui, Arthur Libault, Ahmed Rachid, M. Haissam Jijakli

**Affiliations:** 1 Laboratory of Integrated and Urban Plant Pathology (LIUPP), Gembloux Agro-Bio Tech, University of Liege, Passage des Déportés 2, 5030 Gembloux, Belgium; arthur.libault@uliege.be (A.L.); mh.jijakli@uliege.be (M.H.J.); 2 Laboratory of Chemistry of Natural Molecules, Gembloux Agro-Bio Tech, University of Liège, Passage des Déportés 2, 5030 Gembloux, Belgium; mmohaddab@uliege.be; 3 Laboratory of Agrifood and Health, Faculty of Sciences and Techniques, Hassan First University of Settat, BP 577, Settat 26000, Morocco; 4 Department of Biology and Ecology, Université Libre de Bruxelles (ULB), 1050 Brussels, Belgium; 5 Laboratory of Innovative Technology, University of Picardie Jules Verne, 33 Rue Saint LEU, 80000 Amiens, France; rachid@u-picardie.fr

**Keywords:** decoupled aquaponics, nutrient limitation, trout aquaculture effluent, secondary metabolism, essential oil modulation

## Abstract

Aquaponic systems are among the sustainable approaches for combining fish farming and plant cultivation and have been proposed as potentially economically viable food production technologies. Their performance depends on the balanced environmental conditions shared by fish, nitrifying bacteria, and plants. This study assessed the performance of a trout-based (*Oncorhynchus mykiss*) decoupled aquaponic system for basil (*Ocimum basilicum* L.) growth and essential oil composition. Two cultivation strategies were compared over 60 days: a non-supplemented system relying exclusively on trout rearing water, and a system supplemented with mineral nutrients formulated according to the recommendations of the Hoagland nutrient solution. Basil grown without mineral supplementation maintained a healthy appearance and stable physiological status, with satisfactory growth, although it remained lower than in supplemented plants. The mineral profile of the plants showed similar nitrate and phosphorus concentrations between non-supplemented and supplemented plants, with nitrate levels of 5.40 ± 0.29 mg g^−1^ and 5.52 ± 0.29 mg g^−1^, respectively, and phosphorus levels of 5.46 ± 0.23 mg g^−1^ and 6.14 ± 0.91 mg g^−1^, respectively. In contrast, potassium concentration was lower in non-supplemented plants (36.89 ± 3.31 mg g^−1^) compared to supplemented plants (55.56 ± 7.16 mg g^−1^). Essential oil yield expressed per cultivated surface area remained comparable between systems, reaching 2.96 and 3.09 mL m^−2^ in the supplemented and non-supplemented systems, respectively. GC–MS analysis revealed that linalool (≈24%) was the predominant compound in both systems. Notably, estragole content was higher in non-supplemented plants (21.35 ± 1.46%) compared to supplemented plants (5.24 ± 0.68%). Overall, trout-based aquaponic systems not only support satisfactory basil growth but also enhance the production of essential oils with desirable aromatic characteristics, representing a sustainable and efficient strategy for aromatic plant cultivation.

## Simple Summary

Aquaponics is a sustainable farming method that combines fish and plant production in the same water system. Fish waste naturally provides nutrients for plant growth, reducing the need for chemical fertilizers. In this study, we investigated whether basil can be successfully grown in a trout-based aquaponic system without adding mineral fertilizers. Basil was cultivated for 60 days under two conditions: with and without mineral supplementation. Plants grown without added fertilizers remained healthy, although their growth was slightly reduced. Interestingly, these plants produced more essential oil and showed higher levels of certain aromatic compounds, which are responsible for basil’s characteristic aroma. These results suggest that trout-based aquaponic systems can produce high-quality basil while reducing external inputs, offering a more environmentally friendly alternative to conventional cultivation.

## 1. Introduction

Soilless agriculture currently represents a credible technical alternative to conventional farming, particularly in contexts characterized by water scarcity, progressive soil degradation, and increasing climate variability [1]. The use of controlled cultivation environments allows for more stable and efficient plant production, while improving resource management and input use efficiency [2]. These systems provide greater control over production factors and reduce dependence on external environmental conditions, thereby addressing increasing requirements for sustainability and agronomic performance [3]. Compared to open-field cultivation, they have demonstrated substantially higher yields, often reaching 200–300% increases for leafy vegetables, while simultaneously reducing water consumption by 80–95% [4]. Among the different forms of soilless agriculture, aquaponics operated under coupled or decoupled configurations occupies a prominent position due to its integrative and circular nature [5,6]. Aquaponic systems combine aquaculture and hydroponic plant production within a single framework [7], incorporating cultivation techniques such as the nutrient film technique (NFT) and deep water culture (DWC) [8,9]. In these systems, nutrients derived from fish excreta are biologically transformed by microbial communities and recycled to provide nutrients for the plants. This biological integration confers strong productive potential for both plant and fish compartments, while contributing to more sustainable resource management [7].

At the global scale, aquaponic systems primarily rely on robust fish species such as tilapia (*Oreochromis niloticus*) and catfish (*Siluriformes*) [10,11]. These species are widely used due to their rapid growth, tolerance to relatively high ammonia concentrations, ability to withstand low dissolved oxygen levels, and resistance to pathogens [12,13,14]. Such characteristics make them well adapted to environmental conditions that support efficient nitrification by microorganisms, enhancing biological stability within the system [15]. These fish species are commonly associated with a wide range of vegetable crops, including tomato [16], lettuce [17], and basil [18]. However, the selection of fish species strongly depends on consumer preferences, regulatory frameworks, economic objectives, and the compatibility between fish species and nitrifying microbial communities [19,20]. Consequently, aquaponic practices show considerable regional variability. In Belgium, particularly in Wallonia, trout is the dominant species in aquaculture and is among the most consumed fish nationally, generating an estimated annual direct revenue of between 1 and 1.3 million euros [21]. Nevertheless, trout has strict environmental requirements, particularly in terms of water quality and temperature, with an optimal thermal range of around 15 °C [22]. This temperature is considerably lower than that required for optimal hydroponic plant growth and for the activity of nitrifying bacteria such as *Nitrosomonas* spp. and *Nitrobacter* spp., whose optimal range is 20–30 °C [23,24]. This physiological incompatibility complicates the integration of trout into coupled aquaponic systems. Consequently, the plant compartment often needs to be separated, and technical compromises are required to reconcile fish welfare with biofilter efficiency. These constraints may reduce nutrient availability for plant growth [25], potentially affecting the physiology and secondary metabolism of medicinal and aromatic plants [26,27]. Among these species, basil is a relevant model crop because it is widely cultivated and valued for its culinary and functional properties [28]. It is also characterized by a diverse phytochemical profile, including linalool, estragole, eugenol, geranial, methyl eugenol, and 1,8-cineole [29].

However, despite growing interest in basil (*Ocimum basilicum* L.) and trout (*Oncorhynchus mykiss*) co-production in aquaponic systems, few studies have explicitly examined the constraints imposed by trout farming on the plant compartment. These constraints include low water temperature, high water renewal rates, and limited nutrient accumulation. Their effects on basil agronomic performance and secondary metabolism remain poorly understood. To address this gap, we compared two cultivation strategies: one relying exclusively on water from a trout-based aquaculture unit and one supplemented with mineral nutrients. This study thus evaluates whether basil production without mineral supplementation can maintain agronomic performance and essential oil quality under trout-based aquaponic conditions.

## 2. Study Location and Components

The experiment was conducted from 14 July to 6 September 2025 in a soilless cultivation greenhouse connected to a recirculating aquaculture system at the Urban Agriculture Research Center in Gembloux (Liège, Belgium). Basil was grown under two cultivation modalities: one supplied exclusively with water from the fish rearing, and the other receiving the same water supplemented with mineral nutrients.

### 2.1. System Setup and Experimental Design

#### 2.1.1. Aquaculture Unit System

**Figure 1:**
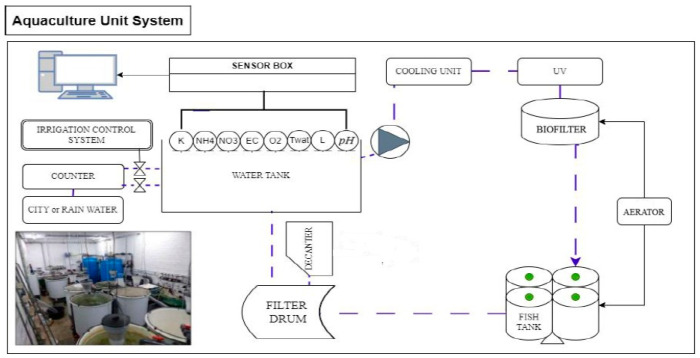
Operational configuration of the recirculating aquaculture system. K: potassium sensor; NH_4_: ammonium sensor; NO_3_: nitrate sensor; EC: electrical conductivity sensor; O_2_: dissolved oxygen sensor; T_wat_: water temperature sensor; L: water level sensor; pH: pH sensor.

The nutrient-rich water used in this study came from a professional aquaculture facility composed of two identical recirculating aquaculture systems (RASs) (Figure 1). Each RAS had a total water volume of 5 m^3^ and included four circular fish tanks. Three of the four tanks were stocked with trout (*Oncorhynchus mykiss*), while one tank remained empty. Fish were stocked at an average density of 1.5 kg m^−3^, corresponding to an initial biomass of 7.5 kg at the beginning of the experiment. Each RAS was equipped with a settling tank, a drum filter with a 60 µm mesh, a moving bed biofilter with a total volume of 2 m^3^ containing 0.6 m^3^ of floating biomedia (specific surface area ≈ 800 m^2^ m^−3^), and a UV sterilization unit. Water overflowing from the fish tanks was collected through a central drain, passed through the drum filter, transferred to a loading tank, and then pumped back into the system using a variable-speed pump. An automatic daily water renewal corresponding to 10% of the total system volume (0.5 m^3^ day^−1^) was implemented. Oxygen was supplied using a 215 W piston compressor and 65 W diaphragm pumps to maintain adequate dissolved oxygen levels in the system. All RAS components could be individually isolated or bypassed to allow maintenance without stopping system operation.

Fish were fed four times per day (7:30 a.m., 11:30 a.m., 3:30 p.m., and 7:30 p.m.) at a feeding rate of approximately 1.65% of total biomass. The diet consisted of B-NATURE GROWER Float pellets (5.5 mm), containing 45% crude protein, 17% crude fat, 1.5% crude fiber, 13.2% ash, and 1.7% phosphorus (P). Water quality parameters, including pH, temperature (T), electrical conductivity (EC), dissolved oxygen (O_2_), and water level, were continuously monitored using electrochemical probes (JUMO GmbH & Co. KG, Fulda, Germany) connected to an automated control system, with SMS alerts triggered when threshold values were exceeded. The pH was maintained at 7.0 ± 0.5, and water temperature was kept at 15 ± 1 °C, in line with the physiological requirements of trout. Ammonium (NH_4_^+^-N), nitrate (NO_3_^−^-N), and potassium (K^+^) concentrations were continuously monitored using a multiparametric ion-selective probe (ISEmax CAS40D, Endress+Hauser, Reinach BL, Switzerland), allowing accurate monitoring of nutrient dynamics within the RAS. Finally, each RAS was connected to the horticultural greenhouse via an underground pipe, supplying the hydroponic units with nutrient-rich aquaculture water.

#### 2.1.2. Hydroponic Unit System

**Figure 2:**
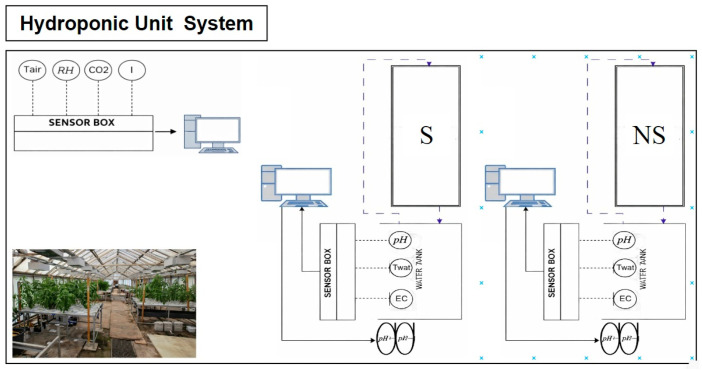
Operational configuration of the greenhouse. S: supplemented system; NS: non-supplemented system; Tair: air temperature sensor; RH: relative humidity sensor; CO_2_: carbon dioxide sensor; I: light intensity sensor; EC: electrical conductivity sensor; T_wat_: water temperature sensor; pH: pH sensor; pH+: peristaltic pump for base dosing; pH−: peristaltic pump for acid dosing.

The greenhouse (Figure 2) was equipped with a Clip Garden fan (HighPro, Madrid, Spain, 5 W) to ensure adequate air circulation and to limit heat accumulation. Lighting relied exclusively on natural sunlight. Light intensity (I) was continuously measured using a quantum PAR sensor (Photosynthetically Active Radiation, Skye Instruments Ltd., Llandrindod Wells, UK) positioned at canopy level. This sensor allowed quantification of photosynthetically active radiation within the 400–700 nm spectral range and enabled precise monitoring of daily light variations. Simultaneously, air temperature (T_air_), relative humidity (RH), and CO_2_ concentration were continuously recorded using environmental sensors based on Vaisala CARBOCAP technology, connected to a data acquisition system (datalogger, Campbell Scientific, Logan, UT, USA). This setup provided real-time, high-resolution monitoring of the greenhouse microclimate.

The experiment was conducted using NFT systems with five growing channels per table. Each channel measured 190 × 12 × 5 cm (length × width × height), providing a total cultivation area of 1.14 m^2^ and 60 planting positions per table, with a spacing of 20 cm between adjacent holes. Plants were grown at a planting density corresponding to one planted hole out of three, using plastic pots filled with rockwool cubes (GRODAN^®^, Roermond, The Netherlands, 77-plug slab), ensuring adequate plant support and uniform exposure of the roots to the nutrient solution. Each NFT system was supplied by a 400 L storage tank located beneath the tables and fed with water originating from trout culture. The water circulated in a closed loop at a constant flow rate of approximately 5 L min^−1^, ensuring a stable nutrient film and adequate root oxygenation. The two reservoirs supplying the experimental tables were equipped with pH probes (SenseCAP S2106, Seeed Studio, Shenzhen, China, coupled with *HI8614LN*, Hanna Instruments, Nușfalău, Romania) and temperature and electrical conductivity probes (DL-CTD10, Sensorea SRL, Antwerp, Belgium), enabling real-time monitoring of physicochemical parameters throughout the cultivation cycle.

#### 2.1.3. Experimental Design

Basil seeds were obtained from Vilmorin (Paris, France). Seeds were sown in a greenhouse using germination trays filled with rockwool and irrigated with water renewed every three days. Transplanting into the experimental greenhouse was carried out 21 days after sowing, simultaneously with the start of trout rearing.

Seedlings were transferred to two independent NFT systems (Experimental Unit), each containing 20 plants. The first system was supplied exclusively with water from an aquaculture unit operated under specific technical trade-offs, notably a reduced temperature (T = 15 ± 1 °C) and a high-water renewal rate (10% per day). The second system received the same water but was supplemented with mineral nutrients using high-purity mineral salts to reproduce the standard Hoagland nutrient formulation [30]. In each system, the total water volume and nutrient concentrations were completely renewed once per week.

Each cultivation modality, supplemented (S) and non-supplemented (NS), was carried out in an independent NFT system within a single production cycle. Therefore, the comparisons reported in this study reflect system-level observations rather than true experimental replications. Individual plants sampled within each system were considered subsamples of their respective system. The pH of the nutrient solution was automatically controlled using peristaltic pumps, which injected a 10% (*v*/*v*) sulfuric acid solution to maintain the target pH range. Nitrogen (N), phosphorus (P), and potassium (K) concentrations were measured on the renewal day by spectrophotometry (IRIS HI801, 340–900 nm, Hanna Instruments). For each system, three water samples were collected from the same basin during each sampling event and analyzed to ensure measurement reliability. These measurements were performed to verify that the S system reached the target Hoagland concentrations and to quantify the nutritional gap relative to the NS system.

### 2.2. Sample Processing

#### 2.2.1. Growth and Agronomic Parameters

At the end of the cultivation period, basil plant height was measured using a graduated measuring tape. Leaf number was counted manually, and leaf area was estimated using the Canopeo online platform (https://canopeoapp.com/). Plants were harvested by cutting at the junction between the stem and the roots. Fresh shoot and root biomass were immediately determined using an electronic precision balance (±0.01 g).

For dry biomass determination and biochemical analyses, five plants per table were randomly selected. Shoots and roots were separated, placed in labeled aluminum containers, and dried in a forced-air oven at 40 °C for five days until constant weight was achieved. Dry shoot and root biomass were then recorded. The dried material from these five plants was pooled and ground into a homogeneous powder. This composite powder was subsequently divided into five subsamples for proline quantification and mineral composition analysis.

The remaining fifteen plants per table were randomly divided into three groups of five plants each. Each group was processed collectively for essential oil extraction and phytochemical analyses, forming one composite sample per group.

#### 2.2.2. Stress Indicators

Plant stress was assessed by determining free proline content following the method described by Bates et al. [31]. Approximately 50 mg of dried plant material was extracted using 3% sulfosalicylic acid. The extract was reacted with ninhydrin and glacial acetic acid and then incubated in a water bath at 90–100 °C for 1 h. The resulting-colored complex was extracted with toluene, and its absorbance was measured at 518 nm. Free proline concentration was determined using a linear calibration curve prepared from standard proline solutions treated under identical conditions (R^2^ = 0.9868), according to the regression equation A = 83.214C − 0.8801, where *A* represents absorbance and *C* the free proline concentration.

In addition, overall photosynthetic efficiency and the specific efficiency of photosystem II (PSII) were measured using a chlorophyll fluorescence fluorimeter (Handy PEA Plus, Hansatech Instruments, King’s Lynn, UK) one hour before harvest. Fully expanded leaves were dark-adapted for 30 min using leaf clips before measurements. A saturating red-light pulse (3000 μmol photons m^−2^ s^−1^, 1 s duration) was applied to induce chlorophyll fluorescence transients. The maximum quantum yield of PSII (Fv/Fm) was calculated as:

$$\frac{Fv}{Fm}=\frac{Fm-F0}{Fm}$$

where Fm maximal fluorescence in the dark-adapted state and F0 represents minimal fluorescence.

The performance index on absorption basis (PI_abs_), which integrates the density of active reaction centers and the efficiency of energy trapping and electron transport, was calculated according to the JIP-test theory as described by Strasser et al. (2004) [32]:

$${PI}_{abs}=\frac{RC}{ABS}\times \frac{\varphi Po}{1-\varphi Po}\times \frac{\psi o}{1-\psi o}$$

where RC/ABS is the density of active reaction centers per absorption, φPo corresponds to Fv/Fm, and ψo represents the efficiency of electron transport beyond QA^−^.

#### 2.2.3. Mineral Profile

The mineral quality of dried basil leaves was assessed by analyzing the concentrations of P, K, calcium (Ca), and magnesium (Mg). Samples consisting of 0.25 g of dry matter (DM) were digested using a microwave digestion system (Multiwave PRO, Anton Paar GmbH, Graz, Austria) with a mixture of nitric acid (HNO_3_) and perchloric acid (HClO_4_) in a 7:1 (*v*/*v*) ratio. Mineral elements were then quantified using ICP-OES (Thermo Fisher Scientific iCAP Plus Series 7000, Waltham, MA, USA) [33].

The N content was determined in dried plant tissue extracts following a colorimetric method [34]. Ground samples (100 mg) were extracted in demineralized water at 45 °C for 1 h. The resulting supernatant was reacted with salicylic acid in concentrated H_2_SO_4_ and subsequently neutralized with 2 N NaOH. Absorbance was measured spectrophotometrically, and nitrate concentration was determined using a calibration curve prepared from nitrate standard solutions (R^2^ = 0.9993), according to the linear regression equation A = 0.0177C − 0.0186, where *A* represents absorbance and *C* the nitrate concentration.

#### 2.2.4. Essential Oil Extraction

Essential oils were extracted by hydrodistillation using a Clevenger-type apparatus [35]. For each system, three composite samples, prepared as described in Section 2.2.1, were processed. Approximately 400 g of fresh plant material was placed in a 5 L round-bottom flask, immersed in water, and heated to gentle boiling. Hydrodistillation was carried out for 5 h, allowing water vapor to transport volatile compounds to the condenser, where the essential oil separated from the aqueous phase. The essential oil obtained from each group was collected in glass vials and stored at 4 °C in the dark until analysis. These extractions represent repeated analytical procedures within a single system rather than independent biological replicates.

#### 2.2.5. Volatile Organic Compound Profile

Essential oil samples were prepared and analyzed by gas chromatography coupled with mass spectrometry (GC–MS) following the method described by Dima Mnayer et al. [36], with slight modifications adapted to this study. Before analysis, the essential oil samples were diluted to 10% (*w*/*w*) in hexane to ensure optimal chromatographic separation and GC–MS compatibility. Analyses were carried out using an Agilent 6890 gas chromatograph coupled to a 5973N mass spectrometer (Agilent Technologies, Santa Clara, CA, USA) equipped with an autosampler. Volatile compounds were separated on a polar VF-WAXms capillary column (20 m × 0.15 mm × 0.15 µm), with Helium was as the carrier gas at a constant flow rate of 0.8 mL min^−1^. The oven temperature was programmed from 60 °C to 250 °C at 10 °C·min^−1^, followed by an 11 min isothermal hold to elute high-boiling compounds. A 1 µL sample was injected in split mode (1:200) with the injector temperature maintained at 250 °C. Mass spectrometry was performed using electron impact ionization, with the ion source and quadrupole temperatures set at 230 °C and 150 °C, respectively. Data was acquired in full-scan mode over a mass range of 30–400 *m*/*z*. Compound identification was achieved by comparing the obtained mass spectra with the NIST 2023 reference database. Retention indices (RIs) were determined using a series of n-alkanes (C7–C30) analyzed under identical chromatographic conditions. Experimental RI values were calculated using the linear Kovats retention index equation (Van den Dool and Kratz) and compared with literature RI values for further confirmation. Phytochemical profile data were processed using Mass Hunter software (version 10.0, Agilent Technologies, Santa Clara, CA, USA) and analyzed with descriptive statistical methods.

### 2.3. Data Analysis

All data were analyzed using Python software (version 3.12). Descriptive statistics were first applied to characterize cultivation conditions, including T_air_, T_wat_, pH, EC, CO_2_ concentration, RH, and I. The same approach was used for agronomic variables (plant weight, leaf number, leaf area, and related parameters), mineral nutrient contents, plant stress indicators, and essential oil yield. Results are expressed as mean ± standard deviation. To further explore differences in the volatile composition between systems, volatile compounds were normalized, log10-transformed, and visualized using a hierarchical clustering heatmap based on Euclidean distance and Ward’s linkage method to compare S and NS systems.

## 3. Results

**Figure A1:**
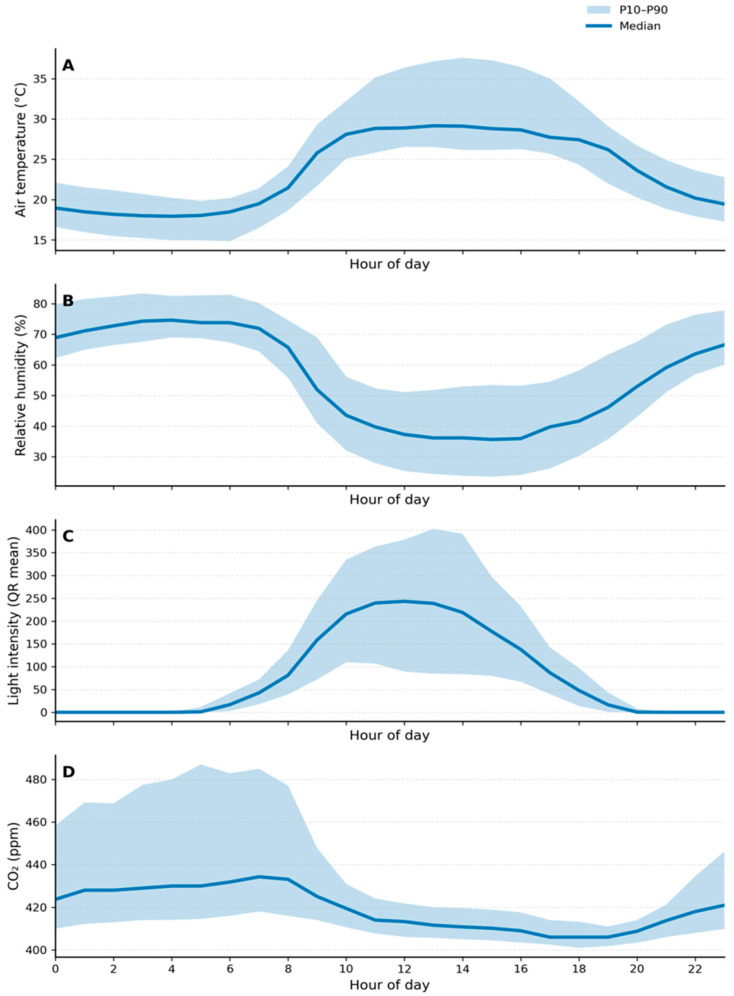
Diurnal variation in (**A**) air temperature, (**B**) relative humidity, (**C**) light intensity, and (**D**) CO_2_ concentration during the experimental period. Solid lines represent median values, while shaded areas indicate the 10th–90th percentile range.

**Figure A2:**
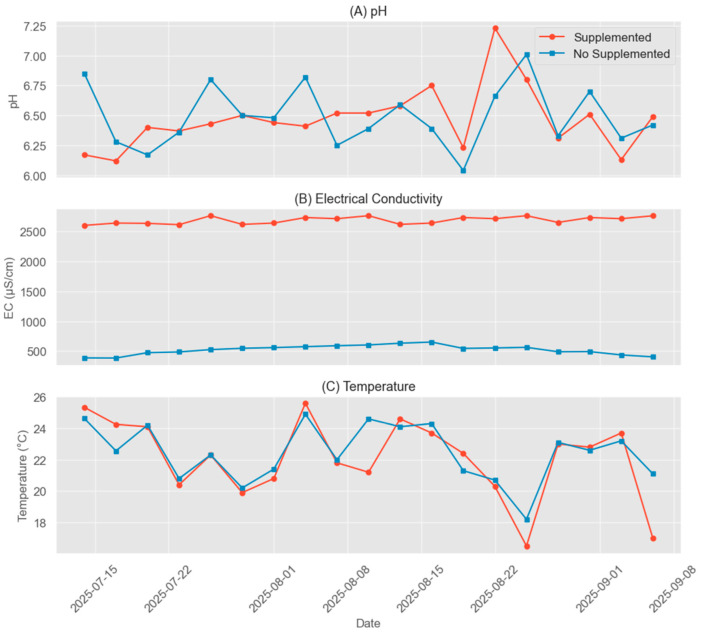
Temporal evolution of (**A**) pH, (**B**) electrical conductivity (EC), and (**C**) water temperature during the experimental period. S: supplemented, NS: non-supplemented.

Detailed information on cultivation conditions during the experimental period (T_air_, RH, light intensity, CO_2_ concentration, T_wat_, EC, and pH) is presented in Appendix A, Figure A1 and Figure A2. Overall, aerial environmental parameters remained stable throughout the experiment. Both the S and NS systems were installed within the same greenhouse and therefore experienced identical aerial environmental conditions. Diurnal air temperature ranged from approximately 17 to 30 °C, with median daytime values between 25 and 28 °C. Relative humidity fluctuated between 35% and 80%, exhibiting a typical inverse relationship with temperature. Photosynthetically active radiation followed a regular daily pattern, reaching maximum values around midday. CO_2_ concentration remained relatively stable over the course of the day, with only minor fluctuations.

In the hydroponic reservoirs, water physicochemical parameters were monitored. pH was consistently maintained between 6.0 and 7.2 in both systems. Water temperature varied between approximately 18 and 26 °C, without consistent differences between systems. In contrast, EC values in the S system ranged from approximately 2600 to 2750 µS cm^−1^, whereas the NS system ranged between approximately 400 and 650 µS cm^−1^. These observations indicate that the primary distinction between systems was related to nutrient concentration rather than environmental variability.

The average nutrient concentrations of system solutions are presented in Table 1. Compared with the S aquaponic system, the NS aquaponic system showed markedly reduced concentrations of N (6.7-fold), P (3.1-fold), and especially K (52.5-fold).

### 3.1. Growth and Agronomic Parameters

**Figure 3:**
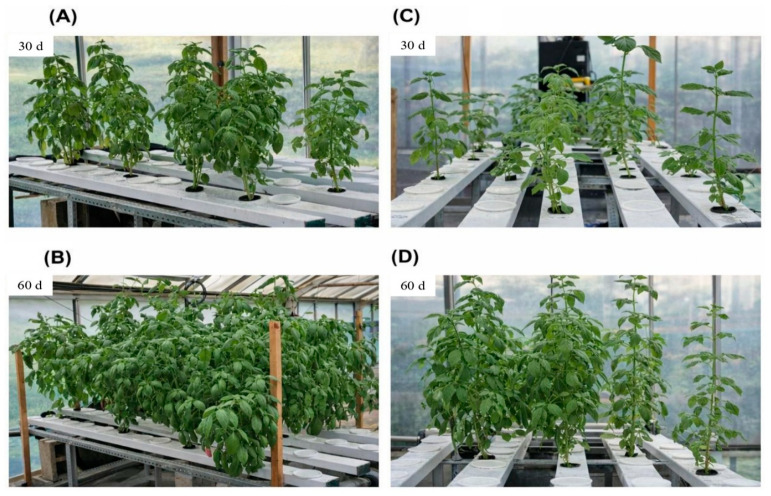
Morphological comparison of basil growth in supplemented (**A**,**B**) and non-supplemented (**C**,**D**) aquaponic systems 30 and 60 days after transplanting.

Basil plants cultivated in both S and NS aquaponic systems exhibited a healthy and relatively uniform appearance (Figure 3), with no apparent signs of severe nutrient deficiency or physiological stress.

**Figure 4:**
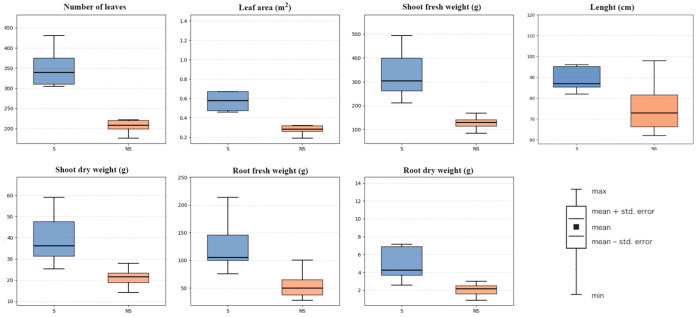
Growth responses of plants under supplemented and non-supplemented systems. The black line in each boxplot indicates the median. S: supplemented; NS: non-supplemented.

At harvest, higher growth values were observed in the S system compared with the NS system (Figure 4). Mean plant height was 90.19 ± 17.29 cm under S conditions compared with 72.60 ± 14.58 cm under NS conditions, representing a 19% reduction. This effect extended to total biomass: fresh shoot and root weights reached 327.96 ± 83.00 g and 122.60 ± 37.74 g in S, compared with 126.12 ± 27.26 g and 53.21 ± 18.88 g in NS, corresponding to reductions of 62% and 57%, respectively. Similarly, dry shoot and root weights decreased from 39.16 ± 9.96 g to 20.91 ± 4.52 g and from 5.53 ± 2.93 g to 2.05 ± 0.62 g, respectively. Leaf-related traits followed a similar pattern, with lower leaf number (213 ± 31 vs. 333 ± 70) and leaf area (0.32 ± 0.15 m^2^ vs. 0.67 ± 0.30 m^2^) observed in the NS system. Nevertheless, canopy architecture remained functional under NS conditions, maintaining sustained photosynthetic activity despite reduced biomass accumulation.

### 3.2. Stress Indicators

**Figure 5:**
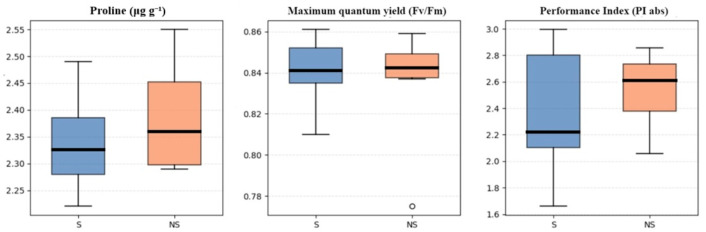
Physiological stress and photosynthetic performance indicators of basil plants under supplemented and non-supplemented aquaponic conditions. The black line in each boxplot indicates the median, and the open circle represents an outlier value. S: supplemented; NS: non-supplemented.

Stress indicators (Figure 5) showed comparable values between the S and NS systems. Free proline concentrations were similar between systems, with mean values of 2.34 ± 0.12 and 2.39 ± 0.11 µg g^−1^ DW for S and NS plants, respectively. Similarly, photosynthetic performance parameters, including the Fv/Fm and PI_abs_, remained stable and were similar between systems, suggesting no marked physiological stress signals under either condition.

### 3.3. Mineral Profile

**Figure 6:**
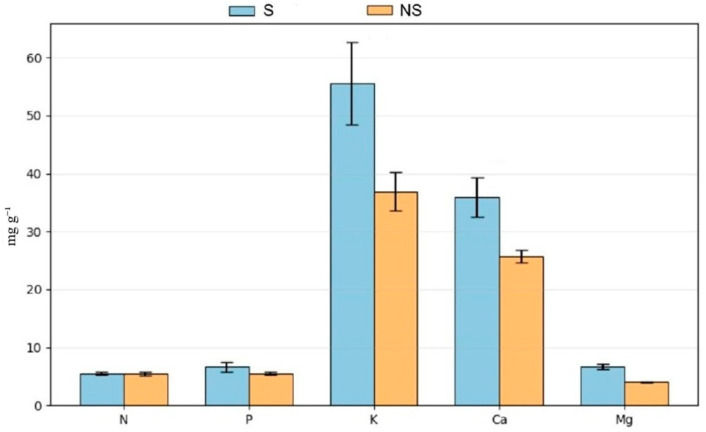
Macronutrients in *O. basilicum* L. leaves grown under supplemented and non-supplemented aquaponic conditions. S: supplemented; NS: non-supplemented; N: Nitrogen; P: Phosphorus; K: Potassium; Ca: Calcium; Mg: Magnesium.

The characterization of leaf mineral status was carried out to evaluate the influence of cultivation practices on the mineral profile of basil. Leaf concentrations of N, P, K, Mg and Ca were quantified and compared between aquaponic systems (Figure 6).

N and P concentrations were identical in S and NS systems. In contrast, lower leaf concentrations of K, Ca, and Mg were observed in the NS system compared with the S system, at 36.89 ± 3.31 vs. 55.55 ± 7.16 mg g^−1^ for K, 25.70 ± 1.16 vs. 35.90 ± 3.39 mg g^−1^ for Ca, and 4.03 ± 0.11 vs. 6.71 ± 0.48 mg g^−1^ for Mg, respectively.

### 3.4. Oil Extraction

The essential oil yield in the S and NS systems (Table 2), when expressed on a fresh matter (FM) basis, was slightly higher in the NS system (1.17 ± 0.13 mL kg^−1^ FM) compared with the S system (0.83 ± 0.02 mL kg^−1^ FM). A similar pattern was observed when yields were expressed on a dry matter (DM) basis, although the between-system variation remained limited (7.07 ± 0.51 mL kg^−1^ DM versus 6.95 ± 0.23 mL kg^−1^ DM for the NS and S systems, respectively). When expressed per unit of cultivated surface, essential oil yields were comparable between systems, with values of 3.09 mL m^−2^ for the NS system and 2.96 mL m^−2^ for the S system.

### 3.5. Volatile Organic Compound Profile

**Figure 7:**
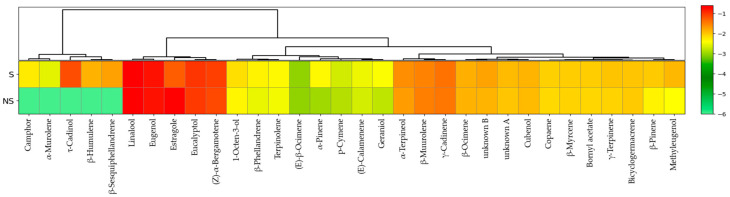
Hierarchical clustering heatmap of normalized VOC abundances highlighting metabolic differences between aquaponic systems. S: supplemented; NS: non-supplemented.

The volatile profile of basil essential oils produced in S and NS aquaponic systems was characterized through the identification and semi-quantification of volatile compounds (Figure 7). Overall, the qualitative composition of the essential oils was largely conserved between systems, with linalool remaining the dominant compound in both conditions (S = 24.715 ± 2.935% and NS = 24.522 ± 0.700%). Most detected compounds exhibited relatively comparable abundance patterns between the two systems, forming clustered groups in the heatmap that reflect a stable core phytochemical profile. Two compounds remained unidentifiable based on the available mass spectral and retention index data and were consequently reported as unknowns (A and B). Within this conserved metabolic background, a clear difference was observed for estragole, which accumulated at much higher levels in the NS system (21.35 ± 1.46%) compared with the S system (5.24 ± 0.68%). The clustering analysis also revealed that several mono- and sesquiterpenes were associated specifically with the S system. Compounds such as camphor, *β*-Humulene, *β*-Sesquiphellandrene, *α*-Murolene, and *τ*-Cadinol were detected exclusively in the essential oils obtained from the S system. These results highlight a shift in the volatile metabolic profile between systems while maintaining the overall aromatic identity of basil essential oil.

## 4. Discussion

Aquaponic systems enable the valorization of fish-derived wastes, including feces and uneaten feed, which constitute a major source of nutrients for plant growth, particularly N and P, both essential for vegetative development [37]. In contrast, potassium is generally present at very low concentrations in aquaponic systems and often represents the main limiting factor for plant growth. Basil, which is highly adaptable to these systems, is widely cultivated for its commercial value and its richness in bioactive compounds that give it recognized medicinal potential [38].

Despite the relevance of the present findings, certain limitations should be acknowledged. The study was conducted over a single production cycle, which may limit the generalization of the results to other climatic conditions or cultivation periods. In addition, the concentrations of Mg, Ca, and micronutrients such as iron (Fe), manganese (Mn), zinc (Zn), copper (Cu), and boron (B) were not analyzed in the aquaponic water solution. This limitation prevents the establishment of a complete mineral balance of the system. Consequently, potential micronutrient deficiencies or imbalances cannot be excluded. Micronutrients play essential roles in photosynthesis, enzymatic regulation, and secondary metabolism. Therefore, they may contribute to the observed differences in growth and the modulation of essential oil composition. Future studies are warranted to include detailed macro- and micronutrient profiling of aquaponic water to clarify the interactions between nutrient availability and plant metabolism in trout-based systems.

### 4.1. Growth and Agronomic Parameters

Agronomic parameters showed contrasting values between systems at the end of the cultivation cycle, including plant height, fresh weight, leaf number, and leaf area, collectively highlighting a distinction between S and NS plants. Similar trends have been reported in aquaponic systems involving different fish species. Research carried out in aquaponic systems based on crayfish and carp demonstrated that basil can maintain satisfactory growth and physiological performance in the absence of mineral supplementation, although biomass accumulation and yield-related parameters remained lower than those observed in S systems [39,40]. Tůmová et al. (2025) [33] reported lower plant biomass for lettuce cultivated in tilapia-based aquaponic systems without fertilization compared with S systems, suggesting that these limitations may be associated with insufficient availability of certain macronutrients. These observations confirm that nutritional availability can represent a major limiting factor for plant growth in aquaponic systems [41]. These studies suggest that mineral supplementation acts more as an optimization lever than as an essential requirement for plant cultivation in aquaponics.

### 4.2. Stress Indicators

Proline content is widely regarded as a reliable indicator of abiotic stress in plants [42,43]. In the present study, free proline levels remained comparable across systems, indicating that nutrient availability in trout-based aquaponic systems remained below the threshold likely to induce physiological stress. Drishya Nishanth (2023) [44] showed that aquaponic systems based on tilapia or catfish could produce basil plants exhibiting lower physiological stress levels than those grown in soil, as evidenced by the absence of a marked increase in proline content. Comparable free proline concentrations were reported by Alizaeh et al. (2025) [6], who observed comparable proline concentrations in watercress (*Nasturtium officinale*) cultivated under tilapia-based aquaponic and mineral hydroponic systems.

Chlorophyll-related indicators showed no variation between nutritional regimes, indicating that the structural and functional integrity of the photosynthetic apparatus was preserved regardless of mineral supplementation. Previous work on lemon basil grown in tilapia-based aquaponic and hydroponic systems reported no differences in chlorophyll content [45]. Similar responses were observed in ‘Primoris’ strawberry plants (*Fragaria × ananassa*) cultivated in an aquaponic system using effluents from thick-lipped grey mullet (*Chelon labrosus*) demonstrated that aquaponically grown plants exhibited different, and often higher, levels of foliar pigments, including chlorophyll, compared to those observed under hydroponic conditions [46]. This finding suggests that, under the specific conditions tested, aquaponic systems were able to sustain physiologically healthy plant growth without additional mineral supplementation.

### 4.3. Mineral Profile

The main macronutrients detected in basil tissues were N, P, K, Ca, and Mg, all essential for plant growth and development [47]. The results indicate that mineral supplementation increased foliar accumulation of K, Ca, and Mg, whereas N and P concentrations remained largely unchanged across systems. This differential response is consistent with the NPK composition of the culture water and reflects the distinct nutritional characteristics of aquaponic systems. In these systems, certain nutrients, particularly N and P, are predominantly supplied through fish farm effluents, whereas K, Ca, and Mg may become limiting in the absence of targeted supplementation [20]. Similar trends have been reported in tomato, basil, and lettuce cultivated under aquaponic and hydroponic conditions, where reduced concentrations of K, as well as certain micronutrients such as Fe and Mn, in aquaponic nutrient solutions represent key limiting factors for plant growth. This potential nutrient limitation likely explains the differences observed between S and NS systems in terms of agronomic performance [48].

### 4.4. Oil Extraction

The results related to essential oil yield indicate that, despite marked differences in vegetative growth and biomass accumulation between systems, moderate nutritional stress maintained relatively stable essential oil productivity. The higher essential oil yield observed on a fresh biomass basis reflects a concentration effect resulting from reduced plant biomass in the NS system. When expressed per cultivated surface area (m^2^), essential oil production was comparable between systems, suggesting that the nutritional regime influenced oil concentration rather than total productivity. These findings indicate that moderate nutrient constraints did not reduce essential oil yield and may have promoted secondary metabolite production. Such a metabolic response likely compensated for biomass differences between systems and enhanced the NS system’s value in terms of product quality and functional potential.

A comparable trend was reported by Roosta et al. (2025) [38], who observed higher essential oil contents in basil cultivated under aquaponic conditions using common carp (*Cyprinus carpio*) as the aquaculture species. However, this aspect remains relatively underexplored in the literature, despite its considerable importance for medicinal and aromatic plants, where the concentration and composition of secondary metabolites are key determinants of agronomic, functional, and commercial value [49].

### 4.5. Volatile Organic Compound Profile

GC–MS analyses showed that basil grown under aquaponic conditions, regardless of the fertilization strategy, exhibited no major changes in the overall aromatic profile of the essential oil. These results indicate that aquaponic cultivation preserves the typical chemical identity of basil, while allowing targeted qualitative and quantitative changes in specific aromatic compounds. Calín-Sánchez et al. (2012) [50] and Tangpao et al. (2022) [51] demonstrated that, in soilless systems, the overall essential oil composition generally remains stable, although specific variations may occur.

However, essential oil composition was affected by the nutritional regime. Reduced mineral availability may induce a shift in secondary metabolism toward the synthesis of specific aromatic compounds rather than biomass accumulation. This response is commonly associated with nutritional modulation [52]. In this study, the markedly higher estragole content observed in NS plants indicates controlled nutritional stress inducing targeted metabolic changes without adversely affecting overall essential oil quality. Estragole is a phenylpropanoid compound involved in plant defense mechanisms, particularly against herbivores and pathogens, and its accumulation is often associated with stress-related activation of the phenylpropanoid pathway [53]. Therefore, its higher concentration under NS conditions likely reflects a metabolic adjustment to mild nutritional imbalance, consistent with a growth defense trade-off under limited mineral availability. This higher estragole proportion coincided with reduced terpenoid diversity, suggesting differential allocation of secondary metabolites depending on nutrient availability, with mineral-limited conditions associated with higher accumulation of phenylpropanoid-derived compounds and lower abundance of several terpenoid compounds. Estragole is known for its moderate antimicrobial [54], antifungal [55], and insecticidal properties [56], which may contribute to the functional value of basil essential oil [57]. However, an excessive increase in estragole concentration may raise toxicological concerns. This compound can be metabolized into 1′-hydroxyestragole, a reactive metabolite capable of forming DNA adducts and inducing hepatocarcinogenic effects in rodents at high doses [58]. Nevertheless, estragole is a major natural constituent of basil essential oil, typically occurring within a range of approximately 20–43% of the total composition [59]. In the present study, estragole concentrations in the NS system remained within this typical range, indicating that the observed increase is within the natural aromatic profile for basil. Nutritionists suggest that moderate nutritional limitations may serve as a metabolic strategy to selectively enhance essential oil quality. Chrysargyris and Tzortzakis (2025) [60] and Kiferle et al. (2013) [61] demonstrated that variations in nutrient solution composition and nutrient availability in soilless systems can influence plant growth and essential oil composition without drastically altering the overall aromatic profile of the plants. These results suggest that water from recirculating aquaculture systems appears to act as a metabolic driver, mainly influencing certain compound groups, especially terpenes.

Accordingly, nutritional management in soilless systems may help modulate basil aromatic composition and guide the accumulation of selected bioactive compounds. However, such modulation should be approached with caution, as alterations in secondary metabolism may also lead to the accumulation of compounds with potential toxicological concerns if their concentrations exceed recommended thresholds [62].

## 5. Conclusions

This study demonstrates that basil can be cultivated in a trout-based decoupled aquaponic system even in the absence of mineral inputs. The exclusive use of aquaculture water enabled the production of plants exhibiting satisfactory physiological status and visual quality, as indicated by low stress indicators and appropriate foliar mineral profiles. These results suggest that trout-derived aquaponic water may cover a large proportion of basil nutritional requirements while supporting essential oil biosynthesis. Essential oil yields in the NS system remained comparable to those obtained under mineral supplementation when expressed per cultivated surface area. However, oil concentration per unit biomass was higher under nutrient-limited conditions. The overall essential oil profile was largely stable between systems, preserving the characteristic basil aromatic chemotype. Notably, a marked quantitative modulation was observed, with a higher estragole content in NS plants, consistent with a potential influence of trout-derived aquaponic water on secondary metabolism and the targeted accumulation of compounds with high functional and commercial value. This modulation likely arises from specific nutritional conditions affecting plant physiology and quality in medicinal and aromatic species.

Future optimization of potentially limiting nutrients, particularly through organic supplementation strategies, could further enhance agronomic performance while maintaining or improving the beneficial effects on essential oil production and composition, and avoiding the adverse effects associated with conventional mineral fertilization.

## Figures and Tables

**Table 1:** Macronutrient concentrations in supplemented and non-supplemented systems measured on the renewal day (mean ± s.d.). S: supplemented; NS: non-supplemented.

System	N (mg L^−1^)	P (mg L^−1^)	K (mg L^−1^)
S	235 ± 3	31 ± 1	210 ± 6
NS	35 ± 1	10 ± 1	4 ± 1

N: Nitrogen, P: Phosphorus, K: Potassium.

**Table 2:** Essential oil yield expressed on a fresh mass, dry mass, and cultivated surface basis in supplemented and non-supplemented systems. S: supplemented; NS: non-supplemented.

System	Oil (mL kg^−1^ FM)	Oil (mL kg^−1^ DM)	Oil (mL m^−2^ S)
S	0.83 ± 0.02	6.95 ± 0.23	2.96
NS	1.17 ± 0.13	7.07 ± 0.51	3.09

FM: fresh matter; DM: dry matter; S: cultivated surface.

## Data Availability

The data presented in this study is available on request from the corresponding author.

## Abbreviations

The following abbreviations are used in this manuscript:

EC	Electrical conductivity
pH	Potential of hydrogen
EO	Essential oil
VOCs	Volatile organic compounds
RI	Retention index
GC–MS	Gas chromatography–mass spectrometry
ICP-OES	Inductively Coupled Plasma–Optical Emission Spectrometry
ISE	Ion-selective electrode
N	Nitrogen
P	Phosphorus
K	Potassium
Ca	Calcium
Mg	Magnesium
NH_4_^+^	Ammonium
FM	Fresh matter
DM	Dry matter
Fv/Fm	Maximum quantum yield of photosystem II
PI_abs_	Performance index on absorption basis
RAS	Recirculating Aquaculture System
NFT	Nutrient Film Technique
DWC	Deep Water Culture
S	Supplemented system
NS	Non-supplemented system
SD	Standard deviation
SE	Standard error
T	Temperature
T_air_	Air temperature
T_wat_	Water temperature
RH	Relative humidity
CO_2_	Carbon dioxide
O_2_	Dissolved oxygen
I	Light intensity
PAR	Photosynthetically Active Radiation
UV	Ultraviolet
